# CRISPR-Cas9 disruption of flavanone 3-hydroxylase produces a green phenotype and alters flavone metabolites in allotetraploid perilla

**DOI:** 10.3389/fpls.2026.1877946

**Published:** 2026-06-15

**Authors:** Shuji Matsushita, Michiharu Nakano, Suguru Chokyuu, Masaki Kurao, Ayane Fujita, Junko Kimura, Chinatsu Nagata, Takeshi Ishikawa, Keita Tamura, Hidemasa Bono

**Affiliations:** 1 Agricultural Technology Research Center, Hiroshima Prefectural Technology Research Institute, Higashi-Hiroshima, Japan; 2 Graduate School of Integrated Sciences for Life, Hiroshima University, Higashi-Hiroshima, Japan; 3 Public Health and Environment Center, Hiroshima Prefectural Technology Research Institute, Hiroshima, Japan; 4 Mishima Foods Co., Ltd., Hiroshima, Japan; 5 Laboratory of Genome Informatics, Graduate School of Integrated Sciences for Life, Hiroshima University, Higashi-Hiroshima, Japan; 6 Laboratory of BioDX, Genome Editing Innovation Center, Hiroshima University, Higashi-Hiroshima, Japan

**Keywords:** anthocyanin, CRISPR-Cas9, flavanone 3-hydroxylase, flavonoid biosynthesis, genome editing, luteolin, *Perilla frutescens*

## Abstract

*Perilla frutescens* var. crispa is a high-value horticultural crop known for its diverse bioactive metabolites, yet the molecular basis underlying its metabolic variation remains poorly understood for targeted metabolic engineering. In this study, we employed CRISPR–Cas9 to disrupt the flavanone 3-hydroxylase gene (*F3H*), a key branch-point enzyme in the flavonoid pathway. We generated stable, T-DNA-free null-segregant lines that exhibited a visible transition from red to green leaves. Metabolite profiling across multiple independently derived edited lines showed that *F3H* disruption markedly reduced anthocyanin accumulation and was consistently associated with increased levels of flavone-related metabolites, including an approximately six-fold increase in luteolin content compared to the wild type. Transcriptome analysis revealed changes in the expression patterns of phenylpropanoid and flavonoid biosynthetic genes consistent with the observed metabolic profiles. In addition, rosmarinic acid content was elevated in the edited lines, suggesting that *F3H* disruption may affect broader phenylpropanoid metabolism. Together, our findings provide functional insights into *F3H* in perilla and highlight the potential of targeted gene editing for modifying metabolite composition in this polyploid crop.

## Introduction

The genus *Perilla* L. (Lamiaceae) comprises annual, self-pollinating plants widely cultivated across East Asia, including China, Korea, and Japan (Nitta et al., 2005). Among the recognized varieties, *Perilla frutescens* var. *frutescens* is primarily grown as an oilseed crop (*egoma*), while *P. frutescens* var. *crispa* is cultivated as a leafy vegetable and culinary herb. The latter is classified into red (*aka-shiso*) and green (*ao-jiso*) types according to leaf and stem pigmentation, a key horticultural trait that directly influences market classification, consumer preference, and end use. In Japan, red perilla is widely utilized for coloring and flavoring pickled plums, with anthocyanins conferring the characteristic red pigmentation (Nitta et al., 2003), and its leaves are processed into dried seasoning products. In contrast, green perilla is marketed as a fresh leafy vegetable, valued for its aroma, taste, and visual appeal in traditional cuisine. Leaf color and phytochemical composition are therefore major determinants of the horticultural and commercial value of perilla cultivars.

*Perilla* contains over 400 identified bioactive compounds, contributing to its value as both a functional food and a medicinal resource (Hou et al., 2022). In traditional Chinese medicine, dried perilla leaves are widely used to treat gastrointestinal disorders, colds, and anxiety (Ahmed, 2019). Notable bioactive constituents include perillaldehyde, a monoterpenoid with reported antidepressant, anticancer, and antibacterial effects (Uemura et al., 2018); anthocyanins, flavonoids with strong antioxidant activity; luteolin, which exhibits both antioxidant and anti-inflammatory effects (Ueda et al., 2002; Jeon et al., 2014; Kim and Lee, 2019a); and rosmarinic acid, particularly abundant in green perilla and known for its potent anti-inflammatory and antioxidant functions (Deguchi and Ito, 2020). Overall, these compounds contribute to health maintenance and disease prevention, making the regulation of their accumulation directly relevant to breeding high-value cultivars and functional food resources.

*P. frutescens* is an allotetraploid species (2*n* = 4*x* = 40), originating from natural hybridization between the diploid species *P. citriodora* (2*n* = 20) and presently unidentified wild relative (Ito and Honda, 1999, 2007; Zhang et al., 2021). This complex polyploid genome is associated with diverse secondary metabolite profiles and poses substantial challenges for genetic analysis. Meanwhile, advances in molecular biological approaches have accelerated research on *Perilla*, leading to the elucidation of secondary metabolic pathways and the cloning of related genes (Gong et al., 1997, 1999; Saito and Yamazaki, 2002). With the rapid development in sequencing technologies, comparative transcriptome analyses of the red and green perilla have been conducted (Fukushima et al., 2015), and more recently, genetic mapping approaches using segregating populations have identified genomic regions associated with leaf pigmentation (Kinoshita et al., 2023; Xie et al., 2025). Furthermore, our group has recently achieved a high-quality reference genome assembly for red perilla (Tamura et al., 2023), increasing the genetic and genomic resources available for breeding.

In recent years, genome editing technologies, particularly CRISPR–Cas9, have been increasingly used to enhance secondary metabolite production in medicinal and functional crops (Das et al., 2024; Rynjah et al., 2025; Sagharyan et al., 2025). In perilla, editing of the *HD3a* gene, a key regulator of photoperiodic flowering, successfully delayed flowering and increased leaf yield (Yun et al., 2023). However, genome editing aimed at modifying the accumulation of bioactive metabolites has not yet been reported (Karthik et al., 2024). Importantly, the broader metabolic consequences of *F3H* disruption within the complex phenylpropanoid and flavonoid networks of an allotetraploid remain poorly understood. In perilla, *F3H* encodes flavanone 3-hydroxylase, which catalyzes the conversion of naringenin to dihydrokaempferol, a key step in anthocyanin biosynthesis (Saito and Yamazaki, 2002). Loss of *F3H* function has been shown to reduce or abolish anthocyanin accumulation in multiple plant species, including strawberry and mulberry, through impaired flavonoid biosynthesis (Dai et al., 2022; Xu et al., 2023). Based on these observations, loss of *F3H* function in red perilla is expected to block anthocyanin accumulation, resulting in a green perilla-like phenotype, and may alter the accumulation of other flavonoid-related metabolites, including luteolin.

In this study, we targeted the *F3H* gene in red perilla using the CRISPR–Cas9 system to generate edited lines with altered pigment and metabolite profiles. Disruption of *F3H* reduced anthocyanin accumulation and was associated with broader changes in flavonoid-related metabolites, including luteolin. Our findings support the feasibility of modifying flavonoid-related traits in perilla using genome editing and provide a useful example for the application of genome editing in polyploid crop species.

## Materials and methods

### Plant materials and growth conditions

For genome editing, *P. frutescens* var. *crispa* cv. ‘Hoko-3’ was used. The transgenic T_0_ plants (primary transformants) generated as described below were acclimatized and subsequently grown in a plant growth chamber. The plants were initially cultivated under long-day conditions (photosynthetic photon flux density, PPFD, 100 μmol m^-^² s^-^¹; 16 h light/8 h dark) at 25 °C for approximately 10 weeks, after which they were shifted to short-day conditions (PPFD 100 μmol m^-^² s^-^¹; 8 h light/16 h dark) to induce flowering and self-pollination for seed production.

A portion of the harvested seeds was grown under long-day conditions in a growth chamber, and the resulting plants were considered the T_1_ generation. Among them, null segregants identified as described below were propagated by excising axillary shoots and rooting them in rockwool to obtain multiple clonal plants. The T_1_ null segregants lacking detectable vector-derived sequences and carrying distinct edited alleles were used for subsequent analyses. To minimize within-line variation, edited lines carrying distinct mutant alleles were clonally propagated and used for subsequent analyses. Multiple independently derived edited lines were analyzed to assess the reproducibility of metabolite changes associated with *F3H* disruption.

These genome-edited lines, together with the commercial green perilla cultivar ‘Ooba Ao-shiso’ (Tohokuseed Co., Utsunomiya, Ltd., Japan) as a phenotypic reference, were transferred to a physically isolated greenhouse and cultivated hydroponically using a standard nutrient solution (OAT House No. 1 and No. 2; OAT Agrio Co., Ltd., Japan). Plants were grown under natural temperature conditions (approximately 20–40 °C) from May to October. All plants were cultivated within the same hydroponic system under uniform nutrient and environmental conditions to minimize environmental variation.

### Vector construction

**Figure 1:**
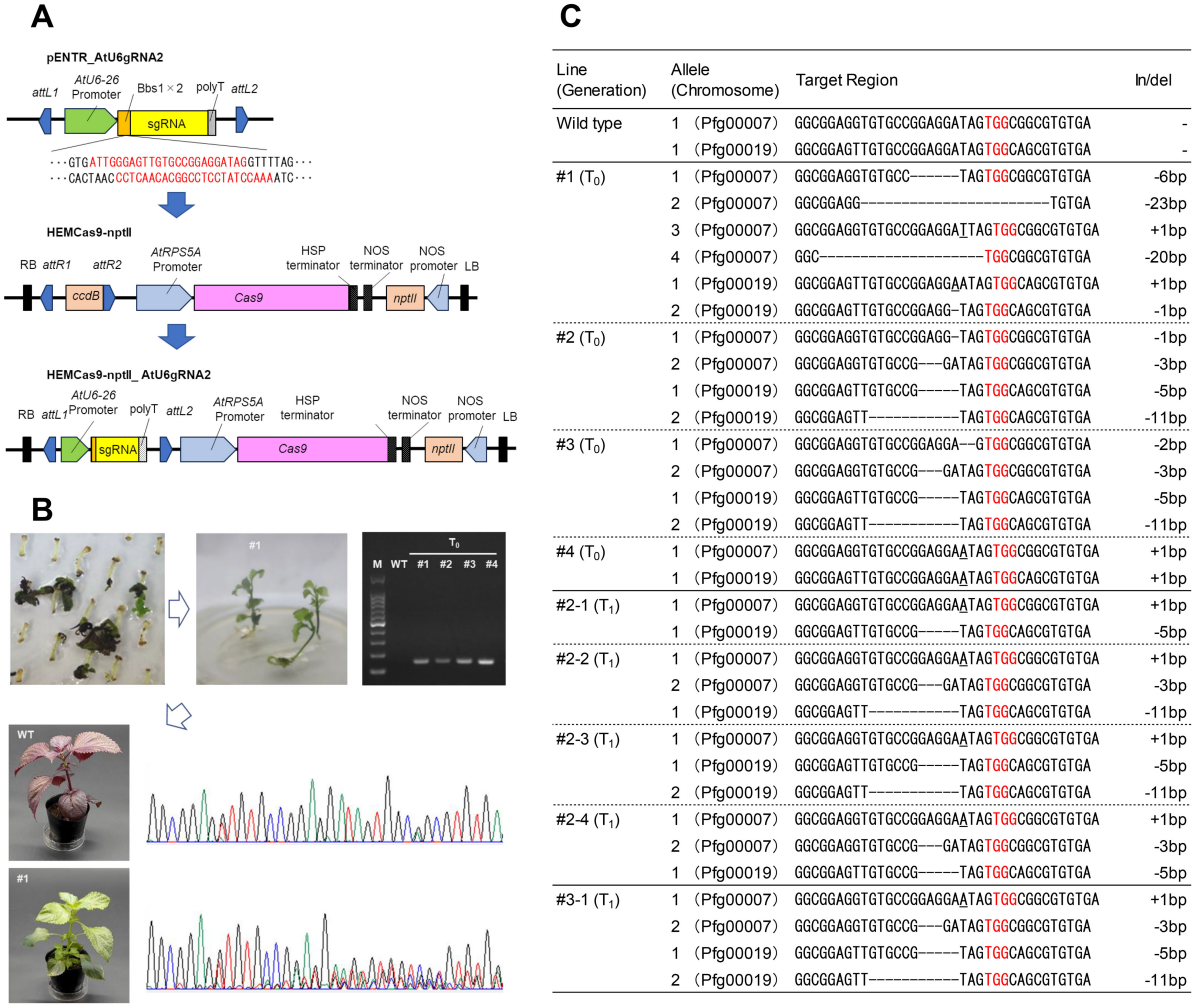
Generation of genome-edited perilla plants. **(A)** gRNA oligos were inserted into pENTR_AtU6gRNA2, and the gRNA cassette was transferred into HEMCas9-nptII by Gateway recombination to generate the genome-editing vector. **(B)** Generation of T_0_ transgenic plants. Shoots were regenerated from hypocotyl explants following *Agrobacterium*-mediated transformation and callus induction, and were subsequently excised for root induction. Rooted plants were acclimated after PCR-based confirmation of transformation, followed by Sanger sequencing analysis. **(C)** Characterization of edited alleles by amplicon sequencing. WT and edited sequences are shown. Red letters indicate the protospacer adjacent motif (PAM) sequences. Dashes (–) represent nucleotide deletions, and underscores (_) denote nucleotide insertions.

At the time of this study, the genome sequence of ‘Hoko-3’ had not been released. Therefore, the *F3H* gene sequence was assembled, and a gRNA was designed within the first exon (**Supplementary Method S1**, **Supplementary Figure 1**). Oligonucleotides synthesized with overhangs compatible with the gRNA and restriction enzyme sites were annealed to form double-stranded DNA and cloned into the entry vector pENTR_ATU6 (Nobusawa et al., 2021) via *BbsI* restriction sites. The gRNA expression cassette was subsequently transferred into HEMCas9-nptII (formerly pGWB401_AtRPS5A-Cas9) (Yamatani et al., 2022) by an LR Clonase reaction (**Figure 1A**). This binary vector was derived from pGWB401, which was developed based on the Gateway binary vector system (Nakagawa et al., 2007). In this construct, Cas9 is driven by the RPS5A promoter and terminated by the HSP terminator. The resulting plasmid was introduced into *Agrobacterium tumefaciens* by electroporation.

### Agrobacterium-mediated transformation

Agrobacterium-mediated transformation experiments were conducted using two *A. tumefaciens* strains (LBA4404 and EHA105) in combination with two hormone conditions (**Supplementary Table 1**). Seeds of *P. frutescens* cv. ‘Hoko-3’ were surface-sterilized by immersion in 70% ethanol for 1 min, followed by treatment with 1% sodium hypochlorite solution for 15 min, and rinsed four times with sterile distilled water. Sterilized seeds were sown on half-strength Murashige and Skoog (MS) medium (Murashige and Skoog, 1962) supplemented with 3% sucrose and solidified with 0.3% gellan gum. After germination, seedlings were grown under long-day conditions (16 h light/8 h dark) at 23 °C for 1 week, and hypocotyls were excised to a length of 0.5–0.8 cm.

*A. tumefaciens* strains were cultured in YEP medium (10 g L^-^¹ yeast extract, 10 g L^-^¹ peptone, and 10 g L^-^¹ NaCl) at 28 °C with shaking at 100 rpm for 24 h. Bacterial cells were collected by centrifugation at 2,000 × g for 10 min using a refrigerated centrifuge (LCX-100, TOMY Seiko Co., Ltd., Tokyo, Japan), and resuspended in an infiltration buffer containing 0.1 mmol L^-^¹ acetosyringone, 10 mmol L^-^¹ MgCl_2_, and 1% Tween 20, and the optical density at 600 nm (OD_600_) was adjusted to 0.1. Hypocotyl segments were immersed in the suspension for 30 min to facilitate infection. Following infection, explants were co-cultivated for 3 days at 23 °C on half-strength MS solid medium supplemented either with 1.0 mg L^-^¹ 2,4-dichlorophenoxyacetic acid (2,4-D) or with 1.0 mg L^-^¹ 6-benzylaminopurine (BA) and 0.1 mg L^-^¹ α-naphthaleneacetic acid (NAA), depending on the experimental condition. Explants were then transferred to a decontamination medium containing 500 mg L^-^¹ cefotaxime and incubated under the same conditions for 7 days. Subsequently, explants were transferred to a selection medium containing 50 mg L^-^¹ kanamycin, with subculture being carried out every 2 weeks for callus induction and selection. After 6–10 weeks, regenerated shoots were excised and transferred to hormone-free half-strength MS solid medium supplemented with 50 mg L^-^¹ kanamycin and 500 mg L^-^¹ cefotaxime for root induction and plant regeneration.

### Confirmation of transformation

PCR analysis confirmed the presence of T-DNA-derived sequences in regenerated plants. Genomic DNA was extracted from leaves using the ISOSPIN Plant DNA Kit (Nippon Genetics, Tokyo, Japan). PCR amplification was performed with primers designed based on the T-DNA sequence (**Supplementary Table 2**) and Quick Taq^®^ HS DyeMix (Toyobo, Osaka, Japan). The PCR conditions were as follows: an initial denaturation step at 95 °C for 5 min, followed by 30 cycles at 95 °C for 30 s, 55 °C for 30 s, and 72 °C for 30 s, with a final extension at 72 °C for 30 s.

### Genome editing validation

Genome editing events were verified by amplicon sequencing. Primers containing linker, tag, and target sequences were used to amplify the target region simultaneously (**Supplementary Table 2**). Genomic DNA extracted from leaves of T_1_ plants was used as template, and PCR was performed under the following conditions: initial denaturation at 95 °C for 5 min, followed by 30 cycles of 95 °C for 30 s, 55 °C for 30 s, and 72 °C for 30 s, with a final extension at 72 °C for 30 s. PCR products were confirmed by electrophoresis, and their concentrations were estimated. All PCR products were pooled, purified with Ampure XP (Beckman Coulter, Brea, CA, USA), and subjected to paired-end sequencing (300 bp) on an Illumina MiSeq platform by Hokkaido System Science Co., Ltd. (Hokkaido, Japan).

### Identification of null segregants

For T_1_ plants, PCR screening was performed as described for T_0_ plants. In five individuals cultivated in an isolated greenhouse (#2-1, #2-2, #2-3, #2-4, and #3-1), the persistence of vector sequences was examined using primers covering the entire vector backbone (Nishihara et al., 2023) (**Supplementary Table 2**). In addition, the RNA sequencing (RNA-seq) data, which were also used for subsequent gene expression analyses, were employed here to examine the presence of vector-derived sequences using a K-mer–based approach. K-mers of length 31 bp were first generated from the vector sequence using Jellyfish (ver. 2.3.0), and perfectly matching reads were extracted from each RNA-seq dataset. K-mers were subsequently re-extracted from the matched reads, and sequences shared with the vector were identified. These sequences were mapped to the reference genome of ‘Hoko-3’ (Pfru_yukari_1.0; https://perilla.annotation.jp/) using Bowtie2 (ver. 2.4.2) (Langmead and Salzberg, 2012). Finally, BLASTn searches against the NCBI nonredundant nucleotide database were conducted to ensure that no nonspecific sequences remained (Camacho et al., 2009).

### Metabolite profiling by HPLC and LC-QTOF/MS

Upper leaves of hydroponically grown plants in an isolated greenhouse were collected from multiple clonal individuals on June 26 and September 11. For the analysis of perillaldehyde and rosmarinic acid, 0.5 g of fresh leaves was chopped and extracted overnight at 10 °C in 5 mL of 80% methanol. The extract was then subjected to sonication for 10 min and centrifugation at 670 × g for 10 min using a refrigerated centrifuge (Model 5420, Kubota Corporation, Tokyo, Japan), and the supernatant was subsequently collected. Anthocyanins were extracted using the same procedure except that 0.5% trifluoroacetic acid (TFA) was used as the extraction solvent. For the analysis of luteolin and its related compounds, 1 g of leaves temporarily frozen at –20 °C was chopped, extracted with 5 mL of 80% methanol, sonicated for 15 min, and centrifuged under the same conditions; the resulting supernatant was collected. All analyses were performed with three biological replicates, each obtained from independently propagated clonal plants representing the same genotype and grown under identical conditions. Quantification of perillaldehyde, rosmarinic acid, anthocyanins, luteolin, and its derivatives was conducted on a Shimadzu high-performance liquid chromatography(HPLC)system (LC-20AT pump, SPD-20AV UV detector, CBM-20A lite system controller; Shimadzu Corporation, Kyoto, Japan) under the conditions listed in **Supplementary Table 3**. Under the analytical conditions used, luteolin and its analogs were not completely resolved by HPLC and were therefore quantified as combined peak areas using luteolin as an external standard. Detailed analysis of luteolin and its analogs was conducted using a liquid chromatography–quadrupole time-of-flight mass spectrometry (LC-QTOF/MS) system, comprising a 1260 Infinity II HPLC system (Agilent Technologies, Santa Clara, CA, USA) coupled with an X500R QTOF mass spectrometer (AB Sciex LLC, Framingham, MA, USA), under the conditions described in **Supplementary Table 4**.

### Transcriptome analysis

Transcriptome analysis was performed to obtain preliminary gene expression profiles in genome-edited plants and to confirm the absence of vector-derived sequences within expressed genes. Total RNA was extracted from leaves harvested on June 18 from hydroponically grown wild type (WT) and three T_1_ plants (#2-1, #2-2, and #2-3) cultivated in an isolated greenhouse using the ISOSPIN Plant RNA Kit (NIPPON GENE Co., Ltd., Tokyo, Japan). RNA-seq libraries were constructed with the VAHTS Universal V8 RNA-seq Library Prep Kit for Illumina. Paired-end sequencing (2 × 150 bp) was carried out on a NovaSeq 6000 platform (Illumina, San Diego, CA, USA) at a depth of 6 GB per sample by Azenta Japan (Tokyo, Japan). Raw reads were processed using Trimmomatic (ver. 0.39) to remove adapter sequences and low-quality bases. Clean reads were mapped to the ‘Hoko-3’ reference genome using HISAT2 (ver. 2.2.1) (Kim et al., 2019b), and transcript abundance was quantified with StringTie (ver. 2.1.7) (Pertea et al., 2015). Because RNA-seq was performed without biological replication, the resulting expression patterns were interpreted descriptively rather than as statistically validated differential expression.

### Off-target analysis

Potential off-target loci of the CRISPR-Cas9 system were predicted using Cas-OFFinder (ver. 2.4.1) (Bae et al., 2014) with up to four mismatches allowed against the Perilla frutescens reference genome (Pfru_yukari_1.0). Predicted candidate off-target sites were further examined using RNA-seq reads obtained from wild-type and genome-edited lines. For each candidate locus, sequencing reads mapped to the corresponding genomic region were extracted using HISAT2, and reads with ambiguous mapping were removed based on mapping quality (MAPQ ≥ 20). To assess sequence variation at each candidate site, aligned reads were parsed using CIGAR-based alignment information to reconstruct nucleotide sequences spanning the predicted off-target regions. Reads showing sequence patterns identical or highly similar to the reference sequence were classified as wild type-like reads, whereas reads containing insertions, deletions, or consistent mismatches within the expected Cas9 cleavage window relative to the reference sequence were classified as edited-like reads. Reads with insufficient coverage of the target region or ambiguous alignment patterns were excluded as noise. Edited-like read frequencies were calculated for each candidate locus, and their reproducibility was evaluated across independently derived genome-edited lines to assess potential off-target editing events.

## Results

### Generation of genome-edited perilla plants

Hypocotyl explants of *Perilla frutescens* ‘Hoko-3’ were subjected to *Agrobacterium*-mediated transformation using two *A. tumefaciens* strains in combination with different hormone conditions (**Supplementary Table 1**). Among the tested conditions, edited regenerants were obtained only using strain EHA105 in combination with BA/NAA-containing medium. From 239 explants cultured under this condition, four PCR-positive regenerants were obtained following callus induction, shoot regeneration, and kanamycin-based selection (**Figure 1B**). Sanger sequencing confirmed the presence of targeted mutations in three regenerated plants. Given the allotetraploid nature of the *P. frutescens* genome, amplicon sequencing was subsequently performed to resolve edited alleles at the targeted loci.

Amplicon sequencing further characterized the edited loci, which were assigned to linkage groups Pfg00007 and Pfg00019 based on the reference genome assembly (Pfru_yukari_1.0*;* Tamura et al., 2023), suggesting that the target sites were located at two distinct genomic loci. In the T_0_ generation, mutations ranging from –23 bp deletions to +1 bp insertions were detected (**Figure 1C**). Line #1 carried four distinct mutations at Pfg00007 and two at Pfg00019, indicative of chimeric editing patterns. Lines #2 and #3 exhibited identical –3 bp deletions at Pfg00007, preserving the reading frame. In line #4, +1 bp insertions were detected at both identified *F3H* loci, resulting in frameshift mutations.

Self-pollination of lines #1–#4 generated T_1_ seeds. Amplicon sequencing of progeny from lines #2–#4 revealed stable inheritance of edited alleles (**Figure 1C**; **Supplementary Table 5**). In line #2, six progeny were analyzed: the –3 bp deletion at Pfg00007 and the –5 bp and –11 bp deletions at Pfg00019 were stably transmitted, whereas the –1 bp deletion at Pfg00007 in the T_0_ generation was replaced by a +1 bp insertion. In line #3, all 14 analyzed progeny consistently inherited the –3 bp mutation at Pfg00007. In line #4, the +1 bp insertions at both identified loci were fixed in the analyzed progeny.

### Metabolite profiling of genome-edited perilla plants

**Figure 2:**
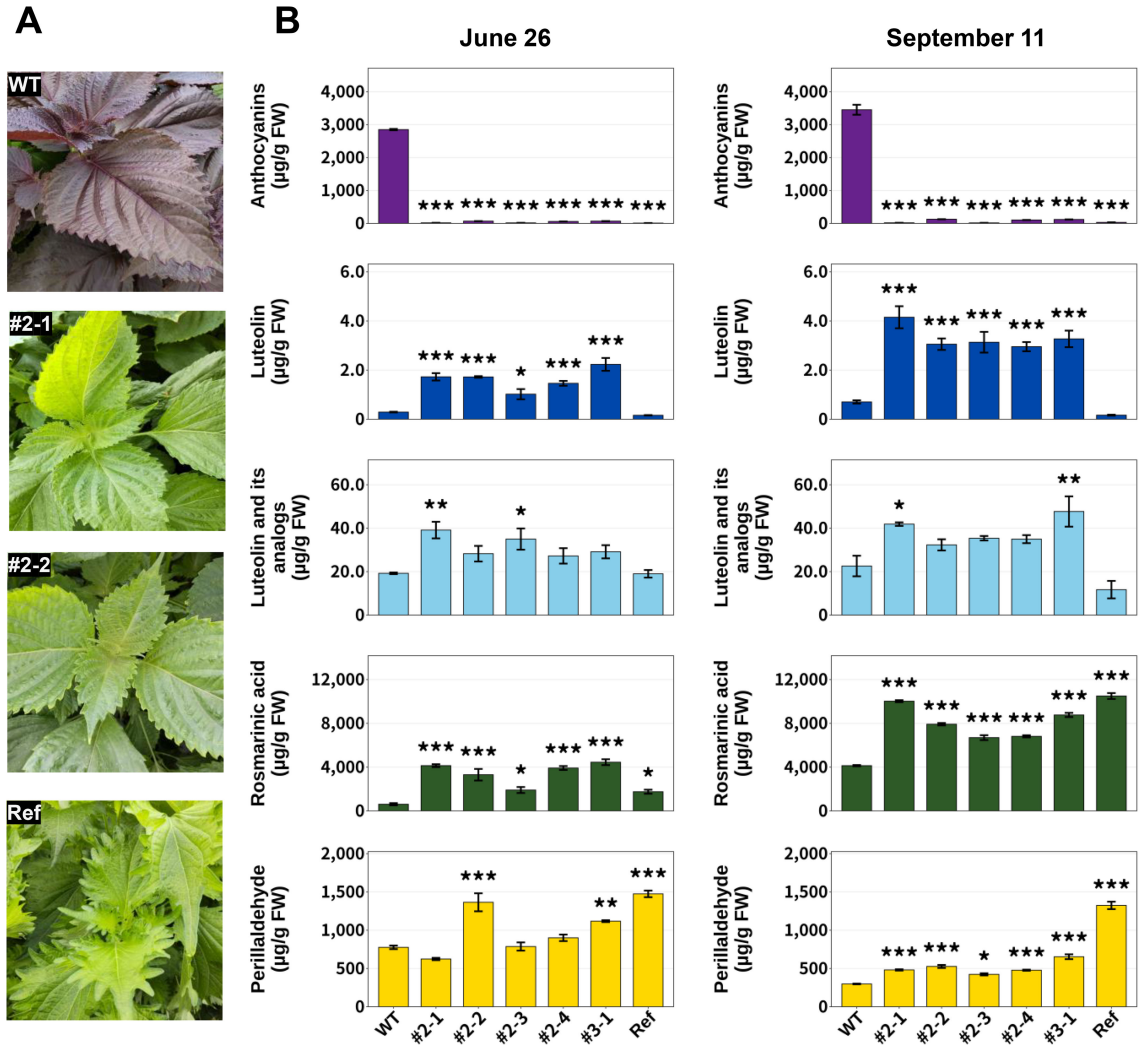
Phenotypic and metabolic profiles of wild type and genome-edited *P. frutescens* lines. **(A)** Representative leaf phenotypes of the samples used for metabolite analysis. Shown are the WT (red perilla ‘Hoko-3’), genome-edited lines (#2–1 and #2-2), and a phenotypic reference (green perilla ‘Ooba Ao-shiso’, hereafter abbreviated as “Ref”). **(B)** Quantification of major metabolites in leaves collected on June 26 (left panels) and September 11 (right panels). Content of five major metabolites—anthocyanins, luteolin, luteolin and its analogs, rosmarinic acid, and perillaldehyde—was determined in WT, genome-edited lines (#2-1, #2-2, #2-3, #2-4, and #3-1), and the green phenotypic reference cultivar. Data are presented as the mean ± SE of three clonally propagated plants per line (*n* = 3). Statistical significance compared to WT was determined by one-way ANOVA followed by Dunnett’s test (*P < 0.05; **P < 0.01; ***P < 0.001). Note that the Y-axis scales are adjusted for each metabolite to highlight differences between lines.

T_1_ individuals from lines #2 and #3 were clonally propagated *in vitro* and subsequently hydroponically cultivated in an isolated greenhouse. A single hydroponic bed was used to grow both the WT (non-edited ‘Hoko-3’) and a phenotypic reference (green perilla ‘Ooba Ao-shiso’) under uniform cultivation conditions. The green perilla cultivar was included primarily as a visual and metabolite reference rather than as a genetically matched control. This experimental design reduced environmental variation among plants and facilitated comparative metabolite analyses among edited genotypes under uniform cultivation conditions. Anthocyanin, polyphenol, and other metabolite contents in the leaves were measured (**Figure 2**).

Genome-edited plants with disrupted *F3H* function predominantly exhibited green leaves, in contrast to the strong red pigmentation along leaf veins observed in WT (**Figure 2A**). Lines carrying the −3 bp deletion showed faint purple coloration along the veins. HPLC analysis of leaves harvested in June and September confirmed that these morphological differences were reflected in the metabolite profiles. Across both sampling dates, WT accumulated 2,814–3,628 µg/g fresh weight (FW) of anthocyanins, whereas edited lines showed drastic reductions in anthocyanin content. Lines with strongly reduced *F3H* activity (#2–1 and #2-3) accumulated only 15–18 µg/g FW, whereas the −3 bp deletion lines (#2-2, #2-4, #3-1) retained slightly higher levels (approximately 56–126 µg/g FW), consistent with residual *F3H* function. Overall, most edited plants had anthocyanin contents below 100 µg/g FW, consistent with their reduced or absent purple pigmentation (**Figure 2B**). Notably, a genotype–phenotype correlation was observed among the edited lines. Lines carrying frameshift mutations at both identified *F3H* loci (#2–1 and #2-3) exhibited the strongest reduction in anthocyanin accumulation, whereas lines carrying the −3 bp in-frame deletion retained partial pigmentation and detectable anthocyanin levels. These observations suggest that the in-frame deletion allele retains partial *F3H* activity.

In WT plants, luteolin levels were only 0.3 µg/g FW in June and 0.7 µg/g FW in September, whereas the five genome-edited lines accumulated 1.0–2.2 µg/g FW and 3.0–4.2 µg/g FW, respectively. For example, line #2–1 accumulated 1.7 µg/g FW in June and 4.2 µg/g FW in September, representing an approximately six-fold increase relative to WT. This trend was consistently observed across all five T_1_ individuals, with significant increases (P < 0.001; except for #2–3 in June, P < 0.05). The combined levels of luteolin-related analogs showed slight variation among edited lines, but no overall substantial differences were observed between the edited lines and the WT. In contrast to luteolin, these analogs did not show a marked increase in leaf tissues.

Rosmarinic acid content was consistently elevated in edited lines relative to WT in both June and September, particularly in #2-1 (up to 10,125 µg/g FW in September), indicating altered metabolite accumulation patterns. Notably, the accumulation of rosmarinic acid in the edited lines was not only significantly higher than in the red WT but also surpassed the levels found in the green phenotypic reference cultivar during the early growth stage. In June, edited lines such as #2–1 and #3–1 exhibited rosmarinic acid levels more than double those of the green phenotypic reference. While the green phenotypic reference showed a substantial increase in rosmarinic acid by September, the edited lines maintained a high accumulation level, reaching a range comparable to or slightly exceeding the green phenotypic reference. Although the genetic background differences between these cultivars preclude direct mechanistic comparisons, this observation indicates that F3H disruption in a red perilla background can drive rosmarinic acid accumulation to levels that match or exceed those of a commercial green cultivar.

While luteolin and rosmarinic acid levels increased from June to September, perillaldehyde exhibited a different pattern. In June, concentrations varied among the lines, whereas in September, an overall decrease was observed across all samples, with levels in the WT dropping to less than half. These results indicate that changes in perillaldehyde accumulation did not parallel those observed for anthocyanins, luteolin, or rosmarinic acid. Seasonal environmental effects may also have contributed to these differences.

### Transcriptomic analysis of flavonoid pathway genes in *F3H*-edited lines

**Figure 3:**
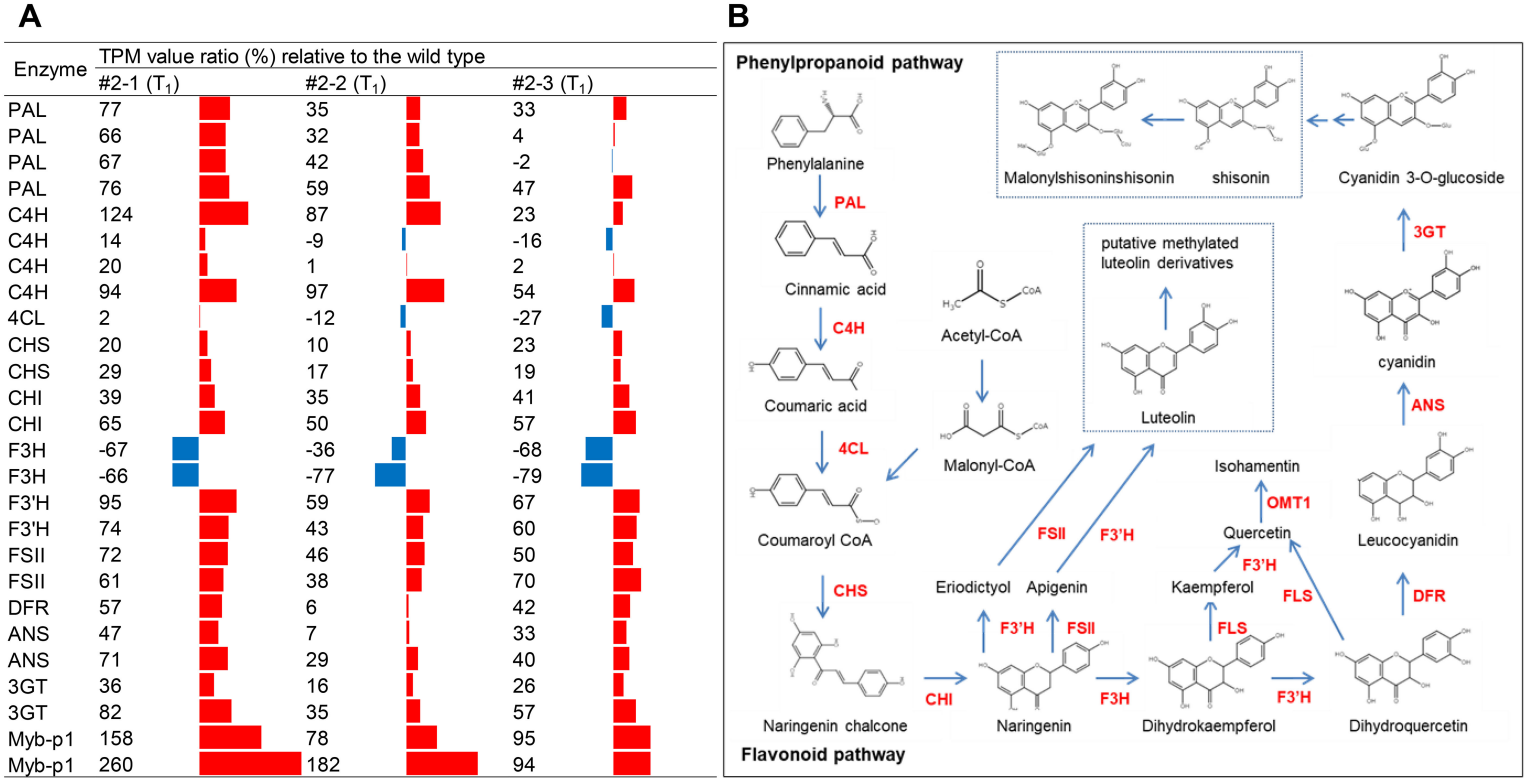
Transcript abundance of flavonoid biosynthesis-related genes and schematic overview of the flavonoid pathway in *P. frutescens*. **(A)** Relative transcript abundance changes (%) of representative biosynthetic genes in T_1_ individuals compared with the WT, based on TPM values obtained from RNA-seq analysis. Numerical values and corresponding bars represent percentage increases (red) or decreases (blue) relative to the WT level (set as 0). Only genes with TPM values ≥ 50 (listed in **Supplementary Table 6**) are shown. **(B)** Schematic representation of the phenylpropanoid and flavonoid biosynthetic pathways. Red-colored abbreviations indicate enzymes corresponding to each catalytic step. PAL, phenylalanine ammonia-lyase; C4H, cinnamate 4-hydroxylase; 4CL, 4-coumarate:CoA ligase; ACC, acetyl-CoA carboxylase; CHS, chalcone synthase; CHI, chalcone isomerase; F3H, flavanone 3-hydroxylase; F3’H, flavonoid 3′-hydroxylase; FSII, flavone synthase II; FLS, flavonol synthase; OMT1, O-methyltransferase 1; DFR, dihydroflavonol 4-reductase; ANS, anthocyanidin synthase; 3GT, anthocyanidin 3-O-glucosyltransferase. Expression values are shown descriptively based on TPM ratios obtained from single RNA-seq samples without biological replication.

Transcriptome analysis revealed that *F3H* expression was markedly reduced in the edited lines (36–79% decrease relative to the WT), whereas genes involved in the phenylpropanoid and flavonoid biosynthetic pathways showed overall increased expression compared with the WT (**Figure 3A**). Upstream phenylpropanoid genes, including *PAL* and *C4H*, tended to be upregulated, while flavonoid biosynthetic genes such as *CHS* and *CHI* exhibited approximately 10–65% higher expression across the edited lines. In addition, *F3’H* and *FSII* showed increases of 43–95% and 38–72%, respectively, relative to the WT. Downstream genes, including *DFR* and *ANS*, were also upregulated by 6–71% compared with the WT. Furthermore, the transcription factor *Myb-p1* exhibited a substantial increase in expression, ranging from 78–260% relative to the WT. These expression changes were observed together with increased luteolin accumulation (**Figure 3B**).

### Generation of genome editing null-segregant plants

To obtain genetically stable genome-edited plants, it was necessary to segregate out genome-editing components in subsequent generations. Segregation of vector-positive and vector-negative progeny within each T_1_ family followed the expected 3:1 Mendelian ratio, as supported by chi-square (χ²) tests (*P* > 0.05) (**Supplementary Table 7**). These results suggest that the T-DNA was inserted at a single genomic locus in each line.

**Figure 4:**
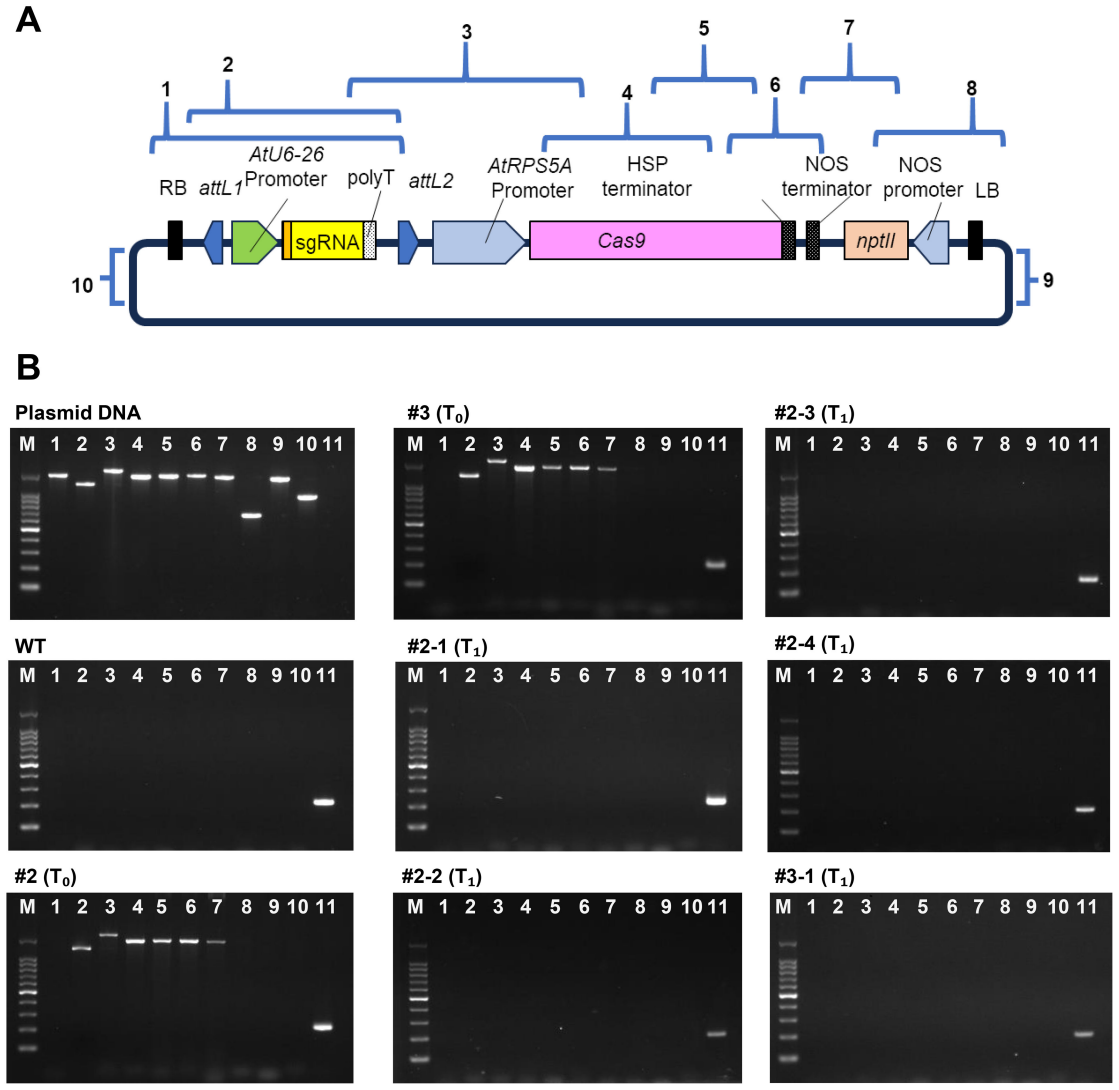
PCR analysis of null segregants. Primer sequences and expected amplicon sizes are listed in **Supplementary Table 2**. **(A)** Schematic diagram of the HEMCas9-nptII_AtU6gRNA2 construct. The plasmid is divided into regions 1–10, of which regions 2–7 are located within the T-DNA region. **(B)** PCR amplification results. Lane M: 100 bp DNA ladder; the upper band corresponds to 1500 bp. Lanes 1–10: PCR amplification of genomic or plasmid DNA corresponding to regions 1–10 in panel **(A)**, performed to assess the presence of vector-derived sequences. Lane 11: PCR amplification of the endogenous *F3H* gene as a positive control for plant genomic DNA.

PCR assays were further performed on T_1_ individuals (#2-1, #2-2, #2-3, #2-4, and #3-1) using primer sets spanning the entire vector backbone (**Supplementary Table 2**). While all target regions were successfully amplified using plasmid HEMCas9-nptII_AtU6gRNA2 as a positive control, no amplification was detected in any T_1_ individual except at the endogenous *F3H* locus, indicating the absence of detectable vector-derived sequences in these plants (**Figure 4**).

To further examine the presence of vector-derived transcripts, RNA-seq data were screened using K-mer analysis (k = 31). A short sequence identical to a vector-derived fragment was detected at a low level in both WT and line #2-1 (WT: GTTAAAATAAGGCTAGTCCGTTATCAACTTG; #2-1: AGCAAGTTAAAATAAGGCTAGTCCGTTATCA). However, these sequences were also detected in WT samples and did not map to the reference genome (Pfru_yukari_1.0). BLASTn analysis revealed high similarity to Escherichia coli strain K12 (AP027461.1), suggesting that these reads likely originated from minor experimental contamination rather than plant-derived transcripts.

Taken together, these results indicate that the analyzed T_1_ individuals (#2-1, #2-2, #2-3, #2-4, and #3-1) represent null segregants in which genome editing components were segregated out, with no detectable evidence of vector-derived sequences or transcripts under the conditions examined.

### Off-target assessment

Candidate loci with up to four mismatches relative to the gRNA sequence were identified from the reference genome. Edited-like reads were detected at one or both intended on-target loci in the edited lines, indicating that edited sequences could be reliably detected by the analysis pipeline (**Supplementary Table 8**). In contrast, edited-like reads were not detected at Pfg00019 (position 42719718) in the #2–2 and #2–3 lines, both of which carried −11 bp deletion alleles at this locus (**Figure 1C**). This observation may reflect reduced recovery of the −11 bp deletion allele and/or reduced transcript abundance in RNA-seq-based analyses.

For candidate off-target loci, the availability of sequencing reads varied among sites. No reads mapping to the corresponding genomic regions were detected for some loci, and analyzable reads were obtained for only a subset of candidate loci. Edited-like reads were generally absent or observed only sporadically at very low frequencies (< 1%), without reproducible enrichment across independently derived edited lines. At Pfg00015 (position 49400795), apparent edited-like frequencies were observed in both wild-type and the #2–2 line; however, these signals were supported by only a very limited number of reads and were not consistently detected among edited lines. Overall, these RNA-seq-based analyses did not provide clear evidence for reproducible off-target editing events associated with F3H genome editing.

## Discussion

In the present study, we generated green perilla lines by genome editing of the *F3H* gene, a key branch-point enzyme in the anthocyanin biosynthetic pathway of red perilla. The edited plants exhibited substantially reduced anthocyanin accumulation together with increased luteolin levels, demonstrating that disruption of a single flavonoid biosynthetic gene can simultaneously alter visible pigmentation and metabolite composition in allotetraploid perilla. These findings support an important role of *F3H* in anthocyanin production in perilla and indicate that its disruption is associated with broader changes in flavonoid and phenylpropanoid-related metabolites. The observed metabolic and transcriptional changes provide physiological insights into *F3H* function in perilla. More broadly, this study demonstrates the applicability of genome editing for functional analysis and metabolite modification in non-model polyploid crops.

Anthocyanins are major pigments distinguishing red and green perilla, and their biosynthetic pathway has been well characterized (Saito and Yamazaki, 2002; Fukushima et al., 2015). Recent genetic studies have identified *F3H*, *C4H*, and the transcription factor *MYB113b* as key determinants of anthocyanin production in perilla (Xie et al., 2025). Consistent with observations in Arabidopsis thaliana, where *F3H* loss-of-function mutants alter flavonoid accumulation (Pelletier et al., 1999), disruption of *F3H* in perilla is expected to block the conversion of naringenin to dihydrokaempferol and consequently reduce anthocyanin biosynthesis (**Figure 3B**). In the present study, reduced anthocyanin accumulation was consistently accompanied by increased luteolin levels across independently edited lines, suggesting that impairment of the anthocyanin branch pathway was associated with altered flavonoid metabolite composition. Although intermediate metabolites and metabolic flux were not directly analyzed, these observations are consistent with altered flavonoid metabolite accumulation patterns following disruption of the *F3H* branch point.

Metabolite profiling further revealed changes extending beyond anthocyanin reduction (**Figure 2**). Under the HPLC conditions used, luteolin and its analogs were detected as partially unresolved peaks, suggesting co-elution of structurally related metabolites. Perilla leaves have been reported to contain multiple luteolin-derived methylated flavonoids in metabolomic analyses (Lee et al., 2017a). LC–QTOF/MS analysis identified an unknown metabolite with a molecular weight consistent with a mono-methylated luteolin derivative. Although methylated luteolin derivatives such as chrysoeriol and diosmetin have previously been reported in perilla seeds and extracts (Lee et al., 2013, 2017b), the detected compound did not match these standards based on retention time and MS/MS fragmentation patterns. Partial similarity to hispidulin was observed; however, the analytical results suggest that the metabolite may represent a distinct luteolin-derived flavone isomer. These observations suggest the presence of a previously uncharacterized luteolin-derived flavone in perilla, providing a basis for future structural and biochemical investigations.

O-methylation of flavonoids is catalyzed by O-methyltransferases (*OMT*s), which often exhibit substrate and regiospecificity, thereby contributing to structural diversification of flavonoid metabolites (Koirala et al., 2016). In perilla, *PfOMT3* has been reported to catalyze methylation of flavonoid substrates (Park et al., 2020). Although luteolin has been suggested as a substrate, the precise methylation position has not been experimentally determined. Multiple *PfOMT3* homologs are present in the perilla genome (**Supplementary Table 6**), and functional diversification among these enzymes could contribute to the production of structurally distinct methylated flavones (Walle, 2009). In addition, enzymes other than *PfOMT3* homologs may also participate in luteolin methylation, as suggested in other plant species (Muzac et al., 2000; Tohge et al., 2007; Cho et al., 2012). The presence of multiple *PfOMT3* homologs suggests that flavonoid methylation in perilla may involve a functionally diversified *OMT* network. However, despite the increase in luteolin accumulation, the combined levels of luteolin-related analogs did not show substantial differences between the edited lines and the WT, suggesting that the accumulation patterns of these related metabolites may be regulated independently under the conditions tested.

Rosmarinic acid levels were also elevated in the edited lines (**Figure 2**). Because flavonoids and other phenylpropanoids share common precursors such as *p*-coumaroyl-CoA (Vogt, 2010; Petersen et al., 2009; Jakovljević et al., 2025), perturbation of branch-point enzymes may influence metabolite accumulation patterns within the phenylpropanoid pathway. The phenylpropanoid pathway is a large carbon flux network connected to the shikimate pathway, in which branch-point regulation is known to determine metabolite distribution (Maeda and Dudareva, 2012). Therefore, the observed metabolite changes are consistent with broader alterations in phenylpropanoid-related metabolism associated with *F3H* disruption. Increased expression of genes such as *PAL* and *C4H* may also be related to altered precursor availability, although direct biochemical and metabolic flux analyses will be required to clarify the underlying mechanisms. Previous studies have similarly reported that perturbation of phenylpropanoid branch points can be accompanied by changes in pathway-wide metabolic and transcriptional states (Besseau et al., 2007). Together with the increased accumulation of luteolin, these observations suggest that editing of a single branch-point enzyme was associated with coordinated changes in multiple classes of specialized metabolites in perilla.

Although metabolite profiling was conducted using a limited number of biological replicates (n = 3), clonally propagated plants grown under identical cultivation conditions were used to minimize environmental variation, particularly differences in light and nutrient availability known to influence flavonoid biosynthesis. In addition, broadly similar metabolic trends were observed across independently derived edited lines carrying distinct mutant alleles. Together, these observations support the association of the detected metabolic changes with *F3H* disruption, although environmental influences cannot be completely excluded.

RNA-seq analysis showed reduced *F3H* transcript levels in the edited lines, which may be associated with nonsense-mediated mRNA decay triggered by frameshift mutations (Drechsel et al., 2013; Tuladhar et al., 2019; Luha et al., 2024). In contrast, several flavonoid biosynthetic genes, including *CHI*, *PAL*, *C4H*, *F3’H*, *FSII*, *DFR*, *ANS*, and *3GT*, showed elevated expression relative to the WT. These genes are normally coordinately activated during anthocyanin biosynthesis in red perilla (Ye et al., 2025; Jiang et al., 2020). Their elevated expression in the edited lines may reflect compensatory transcriptional responses associated with reduced anthocyanin accumulation. In particular, the increased expression of upstream phenylpropanoid pathway genes such as *PAL* and *C4H* may be associated with altered phenylpropanoid metabolism following *F3H* disruption. This interpretation is consistent with previous reports showing that modification of branch-point enzymes in the phenylpropanoid pathway can influence pathway-wide metabolic states (Besseau et al., 2007).

Interestingly, increased expression of *Myb-p1* was accompanied by elevated *DFR* and *ANS* expression despite the marked reduction in anthocyanin accumulation. Previous studies in red perilla suggested that *Myb-p1* is involved in anthocyanin biosynthesis (Gong et al., 1999b), and MYB-related transcription factors are known to coordinately regulate downstream anthocyanin biosynthetic genes, including *DFR* and *ANS*, in various plant species (Dubos et al., 2010; Li et al., 2022; Menconi et al., 2023). The concurrent upregulation of *Myb-p1*, *DFR*, and *ANS* observed in the edited lines is therefore consistent with altered transcriptional regulation of flavonoid biosynthetic genes associated with *F3H* disruption. Although the precise regulatory mechanism remains unclear, these observations suggest that impairment of anthocyanin accumulation can influence transcriptional regulation within the flavonoid biosynthetic pathway.

Because the RNA-seq analysis lacked biological replication, these observations should be interpreted primarily as descriptive transcriptional trends rather than definitive evidence of differential expression. Nevertheless, broadly similar expression patterns were observed across independently derived edited lines carrying distinct mutant alleles, supporting an association between these transcriptional changes and *F3H* disruption rather than line-specific effects.

Genome editing enables the generation of allelic variation that is difficult to achieve through conventional breeding. In perilla, *F3H* exhibits incomplete dominance, and conventional crosses typically produce intermediate phenotypes (Kinoshita et al., 2023; Xie et al., 2025). Notably, the −3 bp deletion allele identified in this study retained partial pigmentation and moderate anthocyanin accumulation, consistent with residual F3H activity. This in-frame mutation resulted in deletion of a single arginine residue while preserving the reading frame, which may explain the intermediate phenotype observed in these lines. This allele may therefore represent a useful genetic resource for fine-tuning pigmentation and flavonoid composition. Molecular analyses further indicated that the edited lines represent null segregants lacking detectable genome editing components, which may facilitate their future use in breeding programs, although regulatory evaluation will depend on jurisdiction.

RNA-seq-based analysis of major candidate loci did not provide evidence for reproducible off-target editing events. While the resolution of RNA-seq may not fully exclude low-frequency or untranscribed genomic alterations, these results are consistent with the absence of detectable recurrent off-target effects under the conditions analyzed. Taken together, these findings suggest that the observed phenotypic changes are primarily associated with targeted disruption of *F3H* loci.

In summary, genome editing of *F3H* in allotetraploid perilla simultaneously altered visible pigmentation and metabolite composition, resulting in reduced anthocyanin accumulation together with increased luteolin and rosmarinic acid levels. These findings indicate that targeted editing of a branch-point enzyme can simultaneously alter visible pigmentation and multiple classes of specialized metabolites in allotetraploid perilla. More broadly, this study provides a framework for future investigations integrating metabolomic, enzymatic, and genetic approaches to better understand and redesign specialized metabolism in allotetraploid non-model species.

## Data Availability

The RNA-seq data generated in this study have been deposited in the DDBJ BioProject database under the accession number PRJDB38062.
