# Supplementary material for: CRISPR-Cas9 disruption of flavanone 3-hydroxylase produces a green phenotype and alters flavone metabolites in allotetraploid perilla
Source: Front Plant Sci. 2026 Jun 15;17:1877946. doi: 10.3389/fpls.2026.1877946 (PMC13311071; doi:10.3389/fpls.2026.1877946)
Supplement: Supplementary file 1 [file SupplementaryFile1.pdf]

Supplementary Information

Title

CRISPR-Cas9 disruption of flavanone 3-hydroxylase produces a green phenotype and alters flavone metabolites in allotetraploid perilla

Authors

Shuji Matsushita, Michiharu Nakano, Suguru Chokyuu, Masaki Kurao, Ayane Fujita, Junko Kimura, Chinatsu Nagata, Takeshi Ishikawa, Keita Tamura, Hidemasa Bono

---

Contents

Supplementary Method: S1

Supplementary Figure: S1

Supplementary Tables: S1–S8

Supplementary Method

Method S1. Target gene sequence identification and gRNA design for CRISPR–Cas9 genome editing in *P. frutescens*.

To perform genome editing using CRISPR–Cas9, it is necessary to design gRNAs specific to the target gene. At the start of this study, the genome sequence of Perilla frutescens var. 'Hoko-3' had not yet been determined; therefore, RNA-seq and Sanger sequencing were conducted to obtain sequence information.

Total RNA was extracted by Bioengineering Laboratory Co., Ltd. (Kanagawa, Japan), and approximately 3 Gb of 151 bp paired-end sequencing data were generated using the Illumina NextSeq 500 platform. The obtained reads were processed with Trimmomatic (ver. 0.39) to remove low-quality reads and adapter sequences (Bolger et al., 2014), followed by de novo assembly using Trinity (ver. 2.14.0) (Grabherr et al., 2011). Coding regions were predicted using TransDecoder (ver. 5.5.0).

The *F3H* CDS (Accession No. AB000286.1) was used as a query for local BLAST searches, and representative sequences showing homology were extracted. The extracted sequences were aligned using ClustalW, as shown in Fig. S1A.

Based on these sequences, primers for sequencing were designed, and Sanger sequencing was performed (Table S2, Fig. S1). From the resulting genomic sequences, the first exon was predicted, and allele information in the allotetraploid genome was inferred from the sequencing traces. Considering that single-nucleotide mismatches located more than 13 bp away from the PAM do not significantly affect genome editing efficiency (Fu et al., 2013; Hsu et al., 2013; Anderson et al., 2015), gRNAs were designed accordingly (Fig. S1). Although multiple gRNA candidates were designed, only Target1 was used in subsequent experiments.

References

Anderson E.M., Haupt A., Schiel J.A., Chou E., Machado H.B., Strezoska Ž. et al. (2015). "Systematic analysis of CRISPR–Cas9 mismatch tolerance reveals low levels of off-target activity." Journal of Biotechnology 211: 56–65. doi:10.1016/j.jbiotec.2015.06.427

Bolger A.M., Lohse M. and Usadel B. (2014). "Trimmomatic: A flexible trimmer for Illumina sequence data." Bioinformatics 30: 2114–2120. doi:10.1093/bioinformatics/btu170

Fu Y., Foden J.A., Khayter C., Maeder M.L., Reyon D., Joung J.K. et al. (2013). "High-frequency

off-target mutagenesis induced by CRISPR-Cas nucleases in human cells." Nature Biotechnology 31: 822–826. doi:10.1038/nbt.2623

Grabherr M.G., Haas B.J., Yassour M., Levin J.Z., Thompson D.A., Amit I. et al. (2011). "Full-length transcriptome assembly from RNA-Seq data without a reference genome." Nature Biotechnology 29: 644–652. doi:10.1038/nbt.1883

Hsu P.D., Scott D.A., Weinstein J.A., Ran F.A., Konermann S., Agarwala V. et al. (2013). "DNA targeting specificity of RNA-guided Cas9 nucleases." Nature Biotechnology 31: 827–832. doi:10.1038/nbt.2647

Supplementary Figure

A

```
AB002816.1    TCCTTAATTAAATAATCCTAGGCAAAAACAACATATATGGCTCAAGCAGTGGGAGTTTCA
'Hoko-3'      ------------------------------------ATGGCTCAAGCAGTGGGAGTTTCA
                                                  ************************

                                          F3H_Seq1F
AB002816.1    CTTACTACACAAGCCAAGGAGAGTACCCTCCACCCCAAATTCATTAGAGATGAAGATGAG
'Hoko-3'      CTTACTACACAAGCCAAGGAGAGTACCCTCCACCCCAAATTCATTAGAGATGAAGATGAG
              ************************************************************

AB002816.1    AGGCCTAAGGTGGCCTACGACCAATTCAGCAGTGAGATTCCGGTGATATCGCTCGCCAGA
'Hoko-3'      AGGCCTAAGGTGGCCTACGACCAATTCAGCAGTGAGATTCCGGTGATATCGCTCGCCAGA
              ************************************************************
                                                  Target site and PAM
AB002816.1    ATCGACGAGAGCGGCGACAAGAGGGCGGAGGTGTGCCGGAGGATAGTGGCGGCGTGTGAG
'Hoko-3'      ATCGACGAGAGCGGCGACAAGAGGGCGGAGTTGTGCCGGAGGATAGTGGCAGCGTGTGAG
              ****************************** *******************.*********
                                                            F3H_Seq2R
AB002816.1    GATTGGGGTATTTTCCAAGTGGTTGATCATGGAGTGCACCCGATGGTCGTTGAAGGGATG
'Hoko-3'      GATTGGGGTATTTTCCAAGTGGTTGATCATGGAGTCGACCCGATGGTCGTTGAAGGGATG
              ***********************************  ***********************

AB002816.1    ACTAGATTAGCTCGAGAATTCTTTGCGTTACCCGATCATGAGAAACTTAGGTTTGATATG
'Hoko-3'      ACTAGATTAGCTCGAGAATTCTTTGCGTTACCCGATCATGAGAAACTTAGGTTTGATATG
              ************************************************************

AB002816.1    TCGGGTGGCAAGAAAGGTGGTTTCATTGTCTCTAGCCATCTTCAG    (Intron)
'Hoko-3'      TCGGGTGGCAAGAAAGGTGGTTTCATTGTCTCTAGCCATCTTCAG    (Intron)
              *********************************************

AB002816.1    GGAGAAGTGGTACAAGACTGGCGCGAAATAGTGACGTATTTTTCGTACCCGATCACGGCC
'Hoko-3'      GGAGAAGTGGTACAAGACTGGCGCGAAATAGTGACGTATTTTTCGTACCCGATCACGGCC
              ************************************************************

AB002816.1    CGGGACTACTCGAGATGGCCGGACAAGCCGGTCGAGTGGCGGGCCGTGACGGAGGGGTAC
'Hoko-3'      CGGGACTACTCGAGATGGCCGGACAAGCCGGTCGAGTGGCGGGCCGTGACGGAGGGGTAC
              ************************************************************

AB002816.1    AGCCAGCTAGTGATGGAATTGGGGTGCAAATTGCTGGAGATTTTATCAGAAGCCATGGGG
'Hoko-3'      AGCCAGCTAGTGATGGAATTGGGGTGCAAATTGCTGGAGATTTTATCAGAAGCCATGGGG
              ************************************************************
                             F3H_Seq1R
AB002816.1    CTTGAAAGAGATGGTCTTTCCAAAGCCTGTGTGGAAATGGATCAGAAAATAGTGGTGAAT
'Hoko-3'      CTTGAAAGAGATGGTCTCTCCAAAGCCTGCGTGGAAATGGATCAGAAAATAGTGGTGAAT
              ***************************** ******************************
```

B

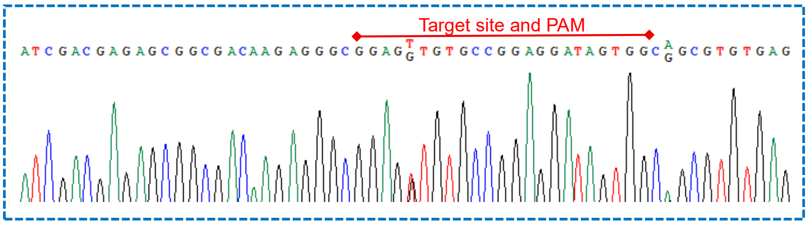

Fig. S1. Sequence organization of the *P. frutescens F3H* gene for gRNA design.

(A) Alignment of the CDS of the perilla *F3H* gene with the 'Hoko-3' sequence reconstructed from RNA-seq data. Red arrows indicate the primers used for sequencing. (B) Sanger sequencing of 'Hoko-3' in the region highlighted by the blue dashed box in panel. Red diamonds indicate the target site and PAM sequence.
